# Association of Matrix Metalloproteinase (MMP) Gene Polymorphisms With Knee Osteoarthritis: A Review of the Literature

**DOI:** 10.7759/cureus.18607

**Published:** 2021-10-08

**Authors:** Christos Milaras, Panagiotis Lepetsos, Dimitra Dafou, Michael Potoupnis, Eleftherios Tsiridis

**Affiliations:** 1 4th Orthopaedic Department, KAT Hospital, Athens, GRC; 2 Biology Department, Aristotle University of Thessaloniki, Thessaloniki, GRC; 3 Academic Orthopaedic Department, Medical School, Aristotle University of Thessaloniki, Thessaloniki, GRC

**Keywords:** articular cartilage, knee, metalloproteinases, single-nucleotide polymorphism, osteoarthritis

## Abstract

Progressive matrix metalloproteinase (MMP)-induced degradation of the extracellular matrix (ECM) of the articular cartilage is one of the major pathogenic osteoarthritis (OA) events. Several single nucleotide polymorphisms (SNPs) in genes encoding MMPs have been identified as affecting MMP expression, production, and enzymatic activity. This study systematically reviews the literature regarding the association between the SNPs of genes encoding MMPs and the risk of knee OA. An electronic search in the PubMed and Web of Science databases from conception to January 2021 was performed addressing studies relating MMPs genetic polymorphisms with the risk of knee OA. We included case-control studies that used validated genotyping methods to detect the SNPs’ association in MMP genes with primary knee OA risk. Ten studies were finally included in this systematic review, evaluating different SNPs in six MMP genes in terms of knee OA pathogenesis: MMP-1 (3 SNPs), MMP-2 (1 SNP), MMP-3 (9 SNPs), MMP-8 (10 SNPs), MMP-9 (6 SNPs), and MMP-13 (1 SNP). Among them, nine SNPs of four MMP genes have been associated with knee OA: (a) MMP-1 -1607 1G/2G (Turkish, Chinese), (b) MMP-3 rs650108, rs650108, rs520540, rs602128, rs679620 (Chinese), (c) MMP-8 rs1940475 and rs376520 (Finnish), and (d) MMP-13 77A/ (rs2252070) (Chinese). The present review summarizes all known SNPs of MMP genes related to a higher risk of knee OA. There are at least nine SNPs in four MMP genes associated with knee OA. No solid correlation between MMP genotype and knee OA phenotype exists. More high-quality studies and modern genetic testing methods are needed to fully elucidate the role of polymorphisms of MMP genes in knee OA pathogenesis.

## Introduction and background

Osteoarthritis (OA) is one of the leading causes of pain and disability worldwide. It is a degenerative disease that mostly affects the articular cartilage, consisting mainly of chondrocytes and extracellular matrix (ECM). The integrity of articular cartilage is maintained by the equilibrium between anabolic and catabolic processes. Any alterations in this balance may contribute to the destructive process within the joint [1]. The progressive degradation of the ECM of the articular cartilage is one of the major pathogenetic events leading to exposure and wear of the subchondral bone, osteophytes formation, and eventually to OA. The etiology of OA is not fully elucidated and may involve complex interactions between genetic and environmental factors [2].

Matrix metalloproteinases (MMPs) are a group of at least 28 proteolytic enzymes, which play an essential role in tissue remodeling and repair during growth and after inflammation. Their primary action is ECM degradation, catabolizing most ECM components such as collagen, laminin, fibronectin, vitronectin, and proteoglycans. MMPs are vital in various physiological and pathological processes such as angiogenesis, cell proliferation, apoptosis, cellular immunity, and cytokine activity [3]. Like all peptidases, MMPs are secreted as inactive proenzymes and activated at the tissue level by the degradation of the N-terminus of the propeptide by other proteases. MMPs bind to membrane receptors, thereby expressing their catalytic activity. MMPs overexpression has been associated with a variety of pathological conditions, such as irreversible tissue degeneration in arthritis and collagen degeneration in cancer patients, predisposing to metastases [4].

MMPs are normally expressed at low levels in the normal joint; however, this expression is augmented in arthritic tissues. Seven MMPs are expressed under various articular cartilage conditions: MMP-1, MMP-2, MMP-3, MMP-8, MMP-9, MMP-13, and MMP-14. Among these molecules, four (MMP-1, MMP-2, MMP-13, and MMP-14) are systematically expressed in adult cartilage participating in tissue metabolism and increasing only in abnormal conditions. The expression of MMP-3, MMP-8, and MMP-9 in cartilage is an exclusive characteristic of pathological conditions. These enzymes are produced either by chondrocytes or synovial cells. Chondrocyte-derived MMPs are considered to be the major catabolic enzymes responsible for the degradation of articular cartilage [5]. MMP-1 and MMP-8 are located in superficial layers of articular cartilage. At the same time, MMP-13 rests in deep layers, which explains that the primary source of MMP-1 and MMP-8 is mainly synovial cells while MMP-8 is mainly produced by chondrocytes. MMP-1 is also produced by activated osteoblasts located within subchondral cysts in OA patients [6]. Collagen is broken down mainly by MMP-13 and secondarily by MMP-2, MMP-7, MMP-8, MMP-9, and MMP-14. The balance between MMPs and their inhibitors (tissue inhibitors of metalloproteinases, TIMPs) is essential for the enzymatic catabolism of articular cartilage [7].

The regulation of MMP production is complemented at the genetic level, as most of the MMPs genes are only expressed when tissue remodeling occurs. MMPs are typically produced by various cells in detectable amounts, but their secretion increases dramatically under the influence of cytokines and growth factors [8]. A single nucleotide polymorphism (SNP) is a type of mutation caused by a nucleotide substitution in a specific gene region. Several SNPs in genes encoding MMPs have been identified, affecting MMP expression, production, and enzymatic activity [9]. However, literature results have shown conflicting results. The aim of this study was to review the literature regarding the association between the SNPs of genes encoding MMPs and the risk of knee OA.

## Review

An electronic search in PubMed and Web of Science databases was performed covering the period from conception until January 2021 to address studies correlating MMPs genetic polymorphisms with knee OA risk. The following keywords were used: (“MMP” OR "MMPs" OR "Metalloproteinase" OR "Metalloproteinases") AND ("SNP" OR "variant" OR “variation” OR "polymorphism" OR “genotype” OR “haplotype”) AND ("osteoarthritis").

Inclusion criteria involved studies (a) using validated genotyping methods to detect the association of SNPs in MMP genes with primary knee OA risk; (b) case-control studies; (c) published in English; (d) with available full text. Exclusion criteria included (a) experimental studies, case reports, systematic reviews, meta-analyses, (b) duplicate studies, (c) studies reporting on rheumatoid or other autoimmune disease arthritis, and (d) studies recording outcomes of other than knee joints with primary OA.

Initially, two investigators (C.M., P.L.) independently screened all the available literature based on the aforementioned inclusion and exclusion criteria. A third investigator resolved any discrepancy in the primary procedure. Two researchers (C.M., P.L.) assessed in duplicate all eligible studies. The following data were extracted for each study: studied genes and SNPs, first author's surname, year of publication, nationality of the studied population, genotyping method, and potential associations with knee OA in both patients and controls.

**Figure 1:**
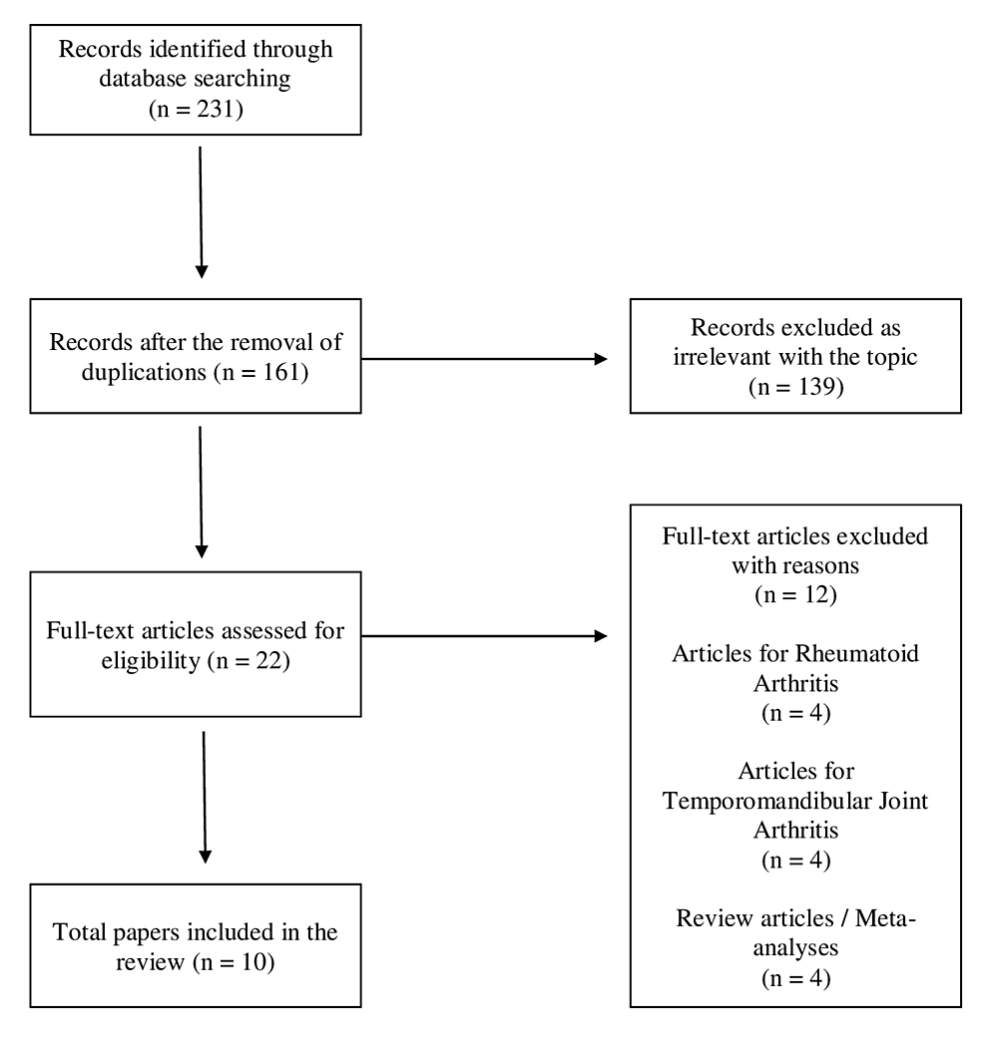
Study flowchart

The primary search revealed 231 available articles. After removing duplicates, 161 references were evaluated. Following the screening of titles and abstracts, 139 were rejected, leaving 22 studies for full-text evaluation. Of these, 12 articles were rejected for various reasons (Figure 1).

Finally, 10 studies were included for qualitative analysis in this review (Table 1) [10-19].

**Table 1:** Characteristics of the included studies MMP: matrix metalloproteinase; NS: not significant; PCR-RFLP: polymerase chain reaction-restriction fragment length polymorphism

STUDY	YEAR	MMP	SNP	ETHNICITY	METHOD	PATIENTS/CONTROLS	ASSOCIATION FINDINGS IN CASE
Barlas [10]	2009	MMP-1	rs1799750	Turkish	PCR/RFLP	157/84	Higher risk in: 1G,1G/1G,1G/2G
			rs243865				NS
			rs3918242				NS
Honsawek [13]	2013	MMP-3	rs3025039	Thai	PCR/RFLP	100/100	NS
Lepetsos [14]	2014	MMP-1	rs1799750	Greek	PCR/RFLP	155/139	Lower risk in 1G/2G in males
Yang [18]	2015	MMP-1	rs1799750	Chinese Han	PCR/RFLP	207/207	NS
			rs498186				NS
			rs1144393				NS
Nakki [15]	2015	MMP-9	rs3933239	Finnish	DNA sequencing	185/895	NS
			rs4810482				NS
			rs3918278				NS
			rs17576				NS
		MMP-8	rs3918261				NS
			rs122749992				NS
			rs3740938				NS
			rs2012390				NS
			rs1940475				NS
			rs7123682				NS
			rs3765620				Higher risk in: G allele
			rs7943404				NS
			rs10895354				NS
Su [19]	2016	MMP-8	rs3740938	Chinese Han	DNA sequencing	300/428	NS
			rs2012390				NS
			rs1940475				NS
			rs11225394				NS
			rs11225395				NS
Guo [12]	2017	MMP-3	rs639752	Chinese	DNA sequencing	100/197	Higher risk in: C/A-C/C vs A/A
			rs650108				NS
			rs520540				Higher risk in: A/G-A/A vs G/G
			rs646910				NS
			rs602128				Higher risk in: G/A-A/A vs G/G
			rs679620				Higher risk in: T/C-T/T vs C/C
			rs678815				NS
			rs522616				NS
Tong [17]	2017	MMP-3	rs639752	Chinese	DNA sequencing	200/231	NS
			rs650108				Higher risk in: G/G vs A/A-A/G
			rs520540				NS
			rs646910				NS
			rs602128				NS
			rs679620				NS
			rs678815				NS
			rs522616				NS
Geng [11]	2018	MMP-1	rs1799750	Chinese	PCR/RFLP	306/401	Higher risk in: 2G allele, 2G/2G
Sun [16]	2019	MMP-13	rs2252070	Chinese Han	DNA sequencing	400/400	Higher risk in: A allele, AA

The included studies evaluated the relation of different SNPs on six MMP genes with knee OA pathogenesis, namely, MMP-1 (3 SNPs), MMP-2 (1 SNP), MMP-3 (9 SNPs), MMP-8 (10 SNPs), MMP-9 (6 SNPs), and MMP-13 (1 SNP). The articles were published between 2009 and 2019. A total number of 2110 patients (range 100-400) and 3082 controls (range 84-895) were evaluated. Six studies evaluated Chinese [11-12,16-19], one study Thailand [13], and three studies Caucasian populations (Greek, Turkish, and Finnish) [10,14-15]. Five studies used the PCR-RFLP method [10-11,13-14,18], and five studies used DNA sequencing techniques [12,15-17,19], as genotyping methods.

MMP-1

Gene encoding is located on the long arm of chromosome 11 (11q22.3). Four studies in the literature have studied the association of -16071G/2G SNP (rs1799750) with knee OA, with conflicting results. The study of Barlas et al., in 2009, first suggested that the 1G allele may predispose to knee OA in the Turkish population [10]. Geng et al. concluded that the 2G/2G genotype and the 2G allele increase knee OA risk in the Chinese Han population [11]. On the other hand, two studies observed no significant association between the rs1799750 SNP and knee OA in Greek and Chinese people, respectively [14,18].

Two more SNPs in the promoter region of the MMP-1 gene have been evaluated in the Chinese Han population [18]. The -755 G/T (rs498186) has been correlated with the formation of a new binding site for the p300 protein [20] while the -519 A/G (rs1144393) polymorphism affects MMP-1 promoter activity [21]. The SNPs mentioned above have not been associated with knee OA in the Chinese Han population [18].

MMP-2

The MMP-2 gene is located on chromosome 16q13-21. The -1306 C/T variant of the MMP-2 gene is found in a core recognition sequence of Sp1 (CCACC box), which abolishes the Sp1-binding site, decreasing MMP-2 promoter activity. Barlas et al. evaluated the SNP mentioned above, concluding that it is not related to knee OA risk in a Turkish population [10].

MMP-3

The gene encoding MMP-3 is located in chromosome 11q22.3. The 5A allele of the -1612 5A/6A polymorphism of the MMP-3 gene is associated with a two-fold higher activity than the 6A allele. The presence of the 6A allele allows the binding of the ZBP-89 repressor that downregulates the expression of the MMP-3 gene. The -1612 5A/6A SNP was not associated with knee OA in the Thai population [13].

Recently, two case-control Chinese studies independently evaluated eight SNPs of the MMP-3 gene (rs639752, rs650108, rs520540, rs646910, rs602128, rs679620, rs678815, and rs522616). Among 200 knee OA patients and 231 controls, Tong et al. detected a weak association of the minor G allele of rs650108 with knee OA risk in a recessive model [17]. In a smaller study group, rs639752, rs520540, rs602128, and rs679620 were associated with an increased risk of knee OA [12].

MMP-8

The MMP-8 gene is located in chromosome 11q22.3. A Finish study analyzed eight SNPs (rs122749992, rs3740938, rs2012390, rs1940475, rs7123682, rs3765620, rs7943404, rs10895354) of the MMP-8 gene in a case-control study. Authors concluded that rs1940475 and rs376520 are associated with knee OA in a Finnish population [15].

MMP-9

The MMP-9 gene is located on chromosome 20q11.2-q13.1 and consists of 13 exons. The -1562 C/T polymorphism at the promoter region is due to a C-T substitution. In vitro, it has been associated with the loss of binding of a nuclear protein causing an increase in transcriptional activity in macrophages [22]. Barlas et al. evaluated this specific SNP, concluding that it is not related to the knee OA risk in the Turkish population [10]. Nakki et al. analyzed five other SNPs of the MMP-9 gene. None of rs3933239, rs4810482, rs3918278, rs17576, and rs3918261 was found to be associated with knee OA in a Finnish population [15].

MMP-13

The MMP-13 gene is located in human chromosome 11q22.2-q22.3. SNPs located in the promoter region can lead to changes in protein expression, structure, and function that predispose to various diseases. The -77A/G polymorphism (rs2252070) results from A by G substitution at position 77 of the gene promoter. The mutant A allele is associated with a two-fold higher transcriptional activity of the MMP-13 gene than that of the G allele [23]. In a case-control study, Sun et al. concluded that the -77A/G polymorphism is associated with a high risk for knee OA and is positively correlated with knee OA severity. The A allele was found to be a vital risk factor for knee OA [16].

Discussion

MMPs are considered the main enzymes responsible for the degradation of ECM components in osteoarthritic articular cartilage. Studies have reported increased MMPs expression levels in all joint tissues of patients with OA [24]. MMPs may exert extremely catabolic actions. Therefore, the normal expressions of the genes encoding them are strictly downregulated during cases of intensive tissue remodeling, occurring during wound healing or embryonic development [25]. MMPs are involved in a plethora of complicated biological actions in both standard and pathological conditions. The present study aimed to review all known studies involving MMPSs in the etiopathogenesis of knee osteoarthritis and found that nine SNPs of the MMP-1, MMP-3, MMP-8, and MMP-13 genes have been associated with a higher risk of knee OA in the Turkish, Finnish, and Chinese populations.

MMP-1 (collagenase 1) is the most widely known protease of the MMP family with the ability to degrade collagen by moving sequentially on the collagen fibers. A single MMP-1 molecule has the ability to cleave multiple collagen chains at different points. MMP-1, expressed in a variety of joint cells, such as chondrocytes, fibroblasts, and osteoblasts is one of the major mediators of ECM degradation of the articular cartilage and irreversible joint destruction in OA. MMP-1 has been found to be overproduced in osteoarthritic rather than normal chondrocytes, demonstrating the important role of MMP-1 in the pathogenesis of osteoarthritis [26]. The expression of MMP-1 is affected by SNPs in the promoter of the MMP-1 gene in chromosome 11. At position -1607 of the promoter, the simple insertion or removal of guanine results in the formation of two alleles: one containing a guanine (1G) base and one containing two guanine (2G) bases. The two guanine bases together with the adjacent adenosine base (5'-GGAA-3’) form a binding site for the Ets family of transcription factors. This binding, in combination with the adjacent binding of the transcription factor AP-1 (activator protein 1) at position -1602, leads to a higher expression of MMP-1 [27].

The -1607 1G/2G SNP (rs1799750) has been extensively studied and associated with various diseases. Expression of MMP-1 is higher in people with the 2G/2G genotype and lower in the 1G/2G and 1G/1G genotypes. Various case-control studies demonstrated that 2G allele carriers are predisposed to multiple cancer types [28] and inflammatory diseases [29]. Besides, the presence of the 2G allele increases AP-1 transcription factor binding and induces higher transcriptional activity than the 1G allele in normal fibroblasts. Four studies have evaluated this SNP association with knee OA [10-11,14,18]. In a Turkish population, the -1607 1G allele predisposed to knee OA [10] while in a Chinese Han population, the -1607 1G allele has a protective role against knee OA [11]. These discrepancies may be attributed to the different populations (Caucasian and Asian), geographical environments, sample sizes, and research designs. Two other MMP-1 SNPs, -755 G/T (rs498186) and -519 A/G (rs1144393) were also evaluated in a Chinese population. However, these SNPs were not associated with an increased risk of knee OA [18].

MMP-2 (gelatinase A) is involved in the catabolism of type IV collagen and is usually expressed early in wound healing [30]. Its expression in normal articular cartilage is low due to the low rate of metabolism of type IV collagen and increases significantly in OA [31]. Once MMP-2 is fully activated, it may contribute to osteoarthritic cartilage degradation [32]. However, the only study that correlated MMP-2 expression with knee OA did not confirm the aetiologic association (Barlas et al.).

MMP-3 is expressed by articular chondrocytes and synoviocytes, participating in synovial inflammation and cartilage turnover in inflammatory joint diseases [33]. MMP-3 breaks down collagen type II, III, IV, IX, and X, specific proteoglycans, fibronectin, laminin, and elastin. Aggrecan is the first molecule to be degraded by MMP-3 [34-35]. In addition, MMP-3 may activate other proteinases such as MMP-1, MMP-7, MMP-9, and MMP-13 [36]. MMP-3 gene expression is primarily regulated during translation from various stimuli such as growth factors and cytokines. The -1612 5A/6A SNP is an MMP-3 gene polymorphism that has been associated with deep venous thrombosis [37] and coronary artery disease [38]; however, this SNP has not been associated with knee OA in a Thai population [13]. A weak association of the minor G allele of rs650108 with knee OA risk in a recessive model [17] has been detected in a Chinese population. In addition, another Chinese study concluded that rs639752, rs520540, rs602128, and rs679620 were associated with an increased knee OA risk [12].

MMP-8 (collagenase 2) is a major ECM degrading protease of, type I, II, and III collagen that enhances ECM breakdown in osteoarthritic cartilage degeneration [39]. MMP-8 participates in the initial steps of collagen degeneration, disrupting triple-helical fibrillar collagen [40]. Intraarticular levels of MMP-8 are higher in OA patients than in controls [24]. In a Finnish population, the MMP-8 rs1940475 and rs376520 polymorphisms have been associated with knee OA pathogenesis [15]. The rs1940475 SNP has also been related to gastric adenocarcinoma [40] and femoral head osteonecrosis [41].

MMP-9 (gelatinase B) is mainly active during embryonic development, playing a role in the angiogenesis of the epiphyseal plate and chondrocyte apoptosis [42]. In articular cartilage, MMP-9 is produced by monocytes and macrophages. Production by chondrocytes is minimal, although these cells appear to play an important regulatory role in the expression of MMP-9 by monocytes [43]. Inhibition of its release by chondrocytes appears to increase in OA, apparently in response to increased MMP-3 activity (possibly through transcriptional regulation) and MMP-13 expression [44]. Serum MMP-9 levels are higher in OA patients, highlighting a possible involvement of the enzyme in the pathogenesis of the disease [26,32]. However, no association has been found between knee OA and the MMP-2 (-1306C/T) and MMP-9 (-1562C/T) gene polymorphisms in a Turkish population [10]. Moreover, none of the rs3933239, rs4810482, rs3918278, rs17576, and rs3918261 SNPs of the MMP-9 gene were related to knee OA in a Finnish population [15]. The rs17576 has been extensively investigated in the literature. It is located in the sixth exon, resulting from A by G replacement at position 836. This SNP affects the substrates binding region of the enzyme MMP-9, substituting an uncharged amino acid (glutamine) from a positively charged amino acid (p.Gln279Arg). This polymorphism potentially alters the protein 3D structure, leading to a change in the enzymatic activity of MMP-9 [45]. It has been associated with asthma [46] and glaucoma [47]. However, no relationship with knee OA was detected.

MMP-13 is by far the most studied MMP in cartilage pathology, as it is considered one of the critical enzymes in knee OA development. It is deemed a major catabolic factor in OA due to its strong ability to break down the articular cartilage type II collagen. MMP-13 has five to 10 times higher activity than MMP-1 in the breakdown of type II collagen [48]. The enzyme also degrades other ECM molecules such as collagen type IV and IX, perlecan, osteonectin, and proteoglycan, playing an important role in ECM metabolism in healthy cartilage [49]. In OA, MMP-13 expression is increased significantly, leading to the higher degeneration of articular cartilage [50]. The -77A/G polymorphism (rs2252070) results from A by G substitution at position 77 of the gene promoter and has been associated with various types of cancer and chronic inflammatory diseases [23]. This SNP has been associated with increased susceptibility and severity of knee OA disease and upregulation of IL-6 and MMP-13 expression levels in a Chinese population [16].

## Conclusions

The present review summarized all known SNPs of MMP genes related to a higher risk of knee OA. Nine SNPs of four MMP genes have been associated with knee OA: (a) MMP-1 -1607 1G/2G (Turkish, Chinese), (b) MMP-3 rs650108, rs650108, rs520540, rs602128, and rs679620 (Chinese), (c) MMP-8 rs1940475 and rs376520 (Finnish), and (d) MMP-13 77A/ (rs2252070) (Chinese). No solid correlation between MMP genotype and knee OA phenotype exists. More studies of high quality and modern genetic testing methods are needed to fully elucidate the role of polymorphisms of MMP genes in knee OA pathogenesis.
